# Efficacy of Combination Therapies on Neck Pain and Muscle Tenderness in Male Patients with Upper Trapezius Active Myofascial Trigger Points

**DOI:** 10.1155/2020/9361405

**Published:** 2020-03-10

**Authors:** Ahmad H. Alghadir, Amir Iqbal, Shahnawaz Anwer, Zaheen A. Iqbal, Hashim Ahmed

**Affiliations:** ^1^Department of Rehabilitation Sciences, College of Applied Medical Sciences, King Saud University, Riyadh, Saudi Arabia; ^2^Department of Building and Real-Estate, Hong Kong Polytechnic University, Kowloon, Hong Kong; ^3^Department of Rehabilitation Sciences, College of Applied Medical Sciences, Najran University, Najran, Saudi Arabia

## Abstract

Myofascial pain syndrome, thought to be the main cause of neck pain and shoulder muscle tenderness in the working population, is characterized by myofascial trigger points (MTrPs). This study aimed to examine the immediate and short-term effect of the combination of two therapeutic techniques for improving neck pain and muscle tenderness in male patients with upper trapezius active MTrPs. This study was a pretest-posttest single-blinded randomized controlled trial. Sixty male subjects with mechanical neck pain due to upper trapezius active MTrPs were recruited and randomly allocated into group A, which received muscle energy technique (MET) and ischemic compression technique (ICT) along with conventional intervention; group B, which received all the interventions of group A except ICT; and group C, which received conventional treatment only. Baseline (Pr), immediate postintervention (Po), and 2-week follow-up (Fo) measurements were made for all variables. Pain intensity and pressure pain threshold (PPT) were assessed by a visual analog scale (VAS) and pressure threshold meter, respectively. All the three groups received their defined intervention plans only. Repeated-measures analysis of variance was used to perform intra- and intergroup analyses. Cohen's *d* test was used to assess the effect size of the applied interventions within the groups. The intergroup analysis revealed significant differences among groups A, B, and C in VAS and PPT at Po (VAS-Po: *F* = 13.88, *p*=0.0001; PPT-Po: *F* = 17.17, *p*=0.0001) and even after 2 weeks of follow-up (VAS-Fo: *F* = 222.35, *p*=0.0001; PPT-Fo: *F* = 147.70, *p*=0.0001). Cohen's *d* revealed a significant treatment effect size within all groups except group C (only significant for VAS-Po–VAS-Pr: mean difference = 1.33, *p* < 0.05, *d* = 1.09); however, it showed a maximum effect size in group A for its variables (VAS-Fo–VAS-Pr: mean difference = 5.27, *p*=0.01, *d* = 4.04; PPT-Fo–PPT-Pr: mean difference = 2.14, *p* < 0.01, *d* = 3.89). Combination therapies (MET plus ICT) showed immediate and short-term (2-week follow-up) improvements in neck pain and muscle tenderness in male patients with upper trapezius active MTrPs.

## 1. Introduction

Working and other age groups are more prone to musculoskeletal disorders that can result in disability [1, 2]. Work-related or entertaining activities that yield repetitive stress on or microtears in a definite muscle or muscle group cause chronic tension in muscle fibers, leading to the formation of trigger points [3]. Impelling activities include holding a telephone receiver between the ear and shoulder to free arms; bouts of bending, sitting with improper back support, inadequate chair arm rest heights; and moving boxes using poor body mechanics [4]. Postural muscles such as the upper trapezius, pelvic girdle muscles, and quadratus lumborum are often affected [5].

A very common painful muscle disorder is caused by myofascial trigger point (MTrP). MTrP is characterized by the presence of a taut band, a hypersensitive painful focus that on compression produces referred sensation, tenderness, motor dysfunction, and autonomic phenomena [6–8]. A trigger point is described as active or latent, depending on its reproduction of clinical symptoms rather than the presence of spontaneous pain [8]. The trigger point that upon compression, either partially or completely, reproduces a familiarized symptom experienced by the patient although it may not be present at the time of examination is considered as an active trigger point; however, the latent TrPs do not reproduce any familiarized clinical presentation experienced by the patients [8–10].

Furthermore, Sonographic methods including sonoelastography, MTrP area, and pulsatility index and mechanosensitivity have been introduced to differentiate between active (higher stiffness and lower PPT) and latent MTrPs [11]. No valid imaging or laboratory tests are available to confirm the presence of MTrPs rather than sonography and palpation methods (flat/pincer palpation) [11]. Palpation method is very common and readily identified by a trained examiner. The diagnosis is made by suspecting the possibility of myofascial pain syndrome (MPS) from the history and then confirming it by identifying the MTrP on a physical examination [10].

Simon [5] suggested that a therapeutic approach that effectively inactivates tender points should constructively impact the trigger points as well. Hence, the management lines have included the application of various electrical modalities, different types of exercises, and manual techniques to produce the immediate effect on reducing neck pain and desensitizing the MTrPs. These applications of hot packs (moist heat), ultrasonic/laser/microwaves/infrared radiation therapies, transcutaneous electrical nerve stimulation, stretching/strengthening exercises, manual techniques (muscle energy technique [MET]/ischemic compression technique [ICT]), and myofascial release techniques (strain-counterstrain [SCS]/integrated neuroinhibitory technique [INIT]) involved in lengthening of shortened or contracted muscle and strengthening of muscles aid the drainage of fluid or blood, improve the range of motion of a stiff joint, and accentuate the relaxation of the contractile component of the muscles [5, 12–15].

The ischemic compression technique involves the direct application of a sustained digital/mechanical pressure over the trigger point with enough strength for a specific time duration, to blockade the blood flow and relieve tension in the area of muscle involved [14, 15]. A widely accepted explanation for the working mechanism behind the therapeutic benefit of ischemic compression is the resurgence of local blood flow upon sudden release of digital pressure, most probably from the spinal reflex mechanism [14, 16]. In addition, the longitudinal elongation of contracted sarcomeres of taut band which results in reducing pain and increasing pressure pain threshold of MTrPs is achieved through the application of ICT as equally achieved by the application of transverse friction massage [5, 16, 17].

The muscle energy technique is an osteopathic treatment technique used to lengthen the soft-tissue tightness [13]. The effective working mechanism of MET follows the postisometric relaxation principle in lengthening the contracted sarcomeres within the taut band that desensitizes the hypersensitive TrPs and, thus, reduces the pain and muscle tenderness in patients with neck pain [13, 17, 18].

Previously, few systematic review studies recommended the application of ICT after dry needling therapy, ICT followed by sustained stretching, and ICT with dry cupping as the most effective treatment option to improve neck pain and inactivate the upper trapezius trigger points [16, 17, 19, 20]. Additionally, researchers advocated that clinical evidence also supports this assumption, especially when the positional release technique is combined with other approaches such as ICT and MET, which have good track records for trigger point deactivation [13]. Therefore, Iqbal et al. [21, 22] and other research associates [14, 15] worked on this assumptions and reported the beneficial effect of the combination of two manual techniques on managing neck pain and upper trapezius muscle tenderness in male patients with MTrPs [21, 22].

MET used alone or in combination with SCS was previously proven effective in immediate, short-term, and long-term management of neck pain caused by active MTrPs of the upper trapezius muscle [14, 15, 22, 23]. However, no studies to date have attempted to reveal the effectiveness of MET combined with ICT for short-term or complete resolution of neck pain and muscle tenderness due to upper trapezius active MTrPs. Therefore, the objective of this study was to determine the immediate and short-term effects of MET combined with ICT for improving neck pain and muscle tenderness in male patients with upper trapezius active MTrPs.

The hypothesis of this study was that the efficacy of MET would be greater when combined with ICT than when used alone to improve neck pain and muscle tenderness in male patients with upper trapezius active MTrPs.

## 2. Material and Methods

### 2.1. Participants

Sixty male subjects with neck pain and muscle aches over the shoulder girdle were screened for inclusion in the study (Shah Physiotherapy Center, Delhi). Those patients who met the inclusion criteria for clinically active palpable MTrPs in a unilateral upper trapezius muscle were recruited. The inclusion criteria were as follows: male subject diagnosed with nonspecific neck pain [24] and muscle tenderness over the upper trapezius muscle due to an active MTrP; age 19–38 years; and presence of a maximum of 1-2 active MTrPs in a unilateral upper trapezius muscle.

Patients were excluded when they were diagnosed with fibromyalgia syndrome according to the American College of Rheumatology criteria [25]; had active MTrPs in the bilateral upper trapezius muscles; had a history of whiplash injury or cervical spine surgery; were diagnosed with cervical radiculopathy or myelopathy determined by their primary healthcare physician; had accepted myofascial pain therapy within 1 month before the study; or showed poor cooperation.

### 2.2. Study Design

This study was a randomized controlled three-arm trial with concealed allocation using http://www.randamization.com to allocate the 60 male participants into three groups. A convenience sampling was used to collect the sample.

### 2.3. Ethical Consideration

Ethical approval was provided by the institutional review board, rehabilitation research chair, King Saud University, Saudi Arabia. This study maintained the human rights, monitored the conduct of appropriate research ethics, and was conducted in accordance with the Declaration of Helsinki (1964). As the Shah Physiotherapy Center did not have any institutional review board, approval for collecting the data was taken from the head of the center and IRB approval was granted by our institution (King Saud University), with whom there was a collaboration agreement. Furthermore, the study was registered and made public on ClinicalTrials.gov PRS (ClinicalTrials.gov Identifier: NCT03840473). A written informed consent was obtained from those who voluntarily participated in this study.

### 2.4. Sample Size

The calculation for sample size to ensure the sufficient power was performed with local software (GPower V. 3.1.9.4). The PPT score with the power of 80% (*F*-test) and a level of significance value 0.05 (2-tailed) were used for estimating the sample size. With effect size of 0.42, 20 participants in each group were required (total sample = 60).

### 2.5. Outcomes

Outcomes were muscle tenderness, i.e., pressure pain threshold and pain intensity assessed by a pressure threshold meter, i.e., pressure algometer (Wagner force dial FDK 20) and a visual analog scale (VAS), respectively. The interclass correlation (0.75–0.89, *F* = 42.55, *p* < 0.01) ranged from good to excellent for the interexaminer reliability of the pressure algometer [26–28]. The VAS is a reliable and valid measurement tool for assessing pain intensity in the clinical setup/research area. The VAS is shown on a100-mm horizontal line marked with two notions on either side. The notion at one end reads “no pain (score 0),” while the other end reads “worst pain imaginable (score 100).” The participants were guided to indicate a visible single spot on this horizontal line expressing their present level of pain [29, 30]. The minimal detectable change (MDC) for PPT and VAS scores was found to be 0.413 kg/cm^2^ and 0.08 cm respectively [31, 32].

### 2.6. Procedures

**Figure 1:**
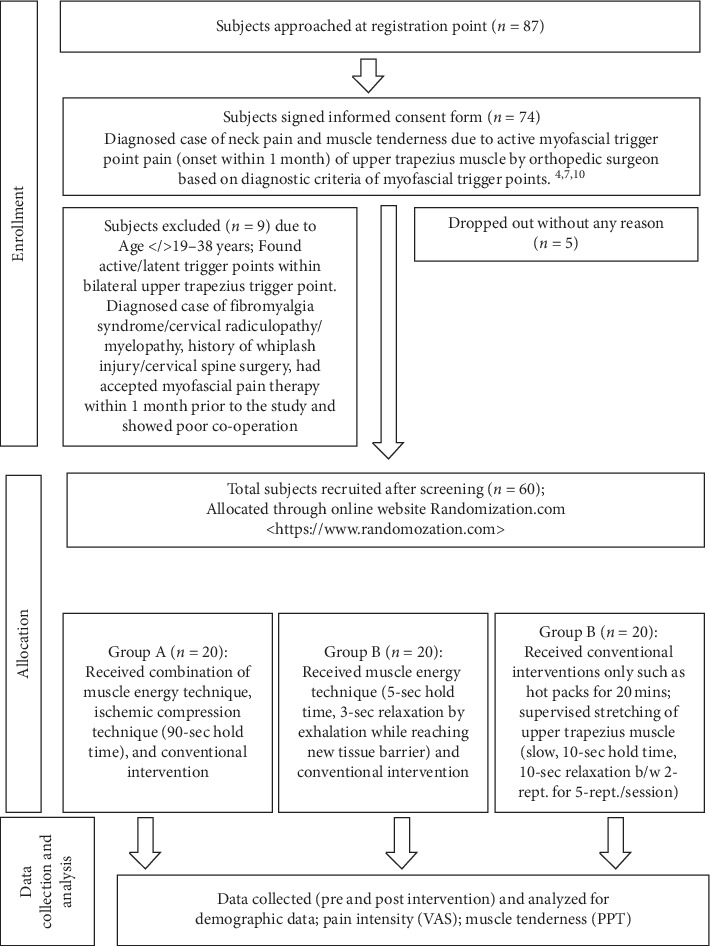
Flowchart of procedures (participant selection, allocation into groups, and data collection).

74 out of 87 subjects were guided to read and sign an informed consent form. Furthermore, 9 subjects did not match the inclusion criteria and 5 subjects dropped out without any reason. 60 subjects who qualified for the inclusion and exclusion criteria were assigned randomly to any of the three groups determined by the online site <http://www.randamization.com>. Irrespective of lab test (not confirmatory test) and MRI test (confirmatory test but much expensive), we follow the standard exploration diagnostic criteria to identify and locate the active MTrPs as described by Simon DG (1999 and 2002), Gerwin RD (1997 and 2014), and Fernández-de-las-Peñas C (2018) in their studies. [4, 8, 10] We considered the following five points to identify and differentiate between active and latent MTrPs: presence of (a) a taut band within the muscle, (b) a hypersensitive tender focus in the taut band, (c) spontaneous pain, (d) local twitch response on snapping palpation, and (e) a referred sensation on palpation [4, 8, 10]. We considered the active MTrPs if they fulfill at least the first three points (a), (b), and (c) and the latent trigger points if they did not fulfill the last two points (d) and (e), thus included and excluded from the study, respectively [8, 10]. PPT and VAS scores were taken just before and 2 minutes after the applied intervention and after 2 weeks of follow-up. Data were collected and sent for analysis. Diagrammatic presentation of study procedures can be understood in Figure 1.

### 2.7. Measurements

The Wagner force dial FDK 20 was used as a pressure algometer to assess the PPT scores of the MTrPs as suggested by Fischer [26]. The trigger point with the lowest PPT value was chosen as a primary trigger point. The subjects were instructed to indicate the sensation of pressure they felt from changing from one of pressure to one of pain by saying “there”/“yes.” Three repeated measurements were obtained by the same assistant, and the mean was used in the analysis. At least a 1-minute gap was added between the two repeated measurements as recommended by Fischer [26]. After taking preintervention data for the PPT, a second application of 2.5 kg/cm^2^ of pressure was applied at the rate of 1 kg/cm^2^ by the physiotherapist while the subjects were stated to rate their pain on the VAS to evaluate local pain evoked by the application of that amount of pressure [29, 30]. All collected data were sent for analysis.

### 2.8. Interventions

The interventions were delivered to all groups only one time. Group A received hot packs (75°C) for 20 minutes and active stretching exercises for upper trapezius muscle (slow, 5 repetitions per session, 10-second hold and 10-second relaxation between two repetitions) followed by ICT (90-second hold) and MET (5-second hold, 3-second relaxation by exhalation while reaching the new barrier). Group B received all the exercises of group A except ICT. Control group C received all the exercises of group B except MET. Active stretching exercises were done by all the participants under the supervision of physical therapist. This approach was standardized for all participants.

For the MET, the patient was in a supine position with the cervical spine in the opposite lateral flexion to the treating part so that the upper trapezius muscle fibers were in a lengthened position [22]. The moderate isometric contraction (approximately 75% of maximal) of the upper trapezius muscles was elicited for a period of 5 seconds followed by 3 seconds of relaxation while reaching the new barrier. The technique was repeated four times in each session. Each subject was placed on a full back supported chair without arm rests and completed the maneuver under the therapist's supervision for active stretching.

For the ICT, the patient was in the supine position with the cervical spine in opposite lateral flexion to the treating part so that the upper trapezius muscle fibers were kept in a lengthened position [21, 26]. The physiotherapist applied gradually increasing pressure to the MTrPs until the subject perceived the first noticeable pain. At that moment, the pressure was maintained until the discomfort and/or pain eased by around 50% as perceived by the patient, at which time the pressure was increased until the discomfort appeared again. This process was maintained for 90 seconds.

### 2.9. Analysis

SPSS version 17.0 software was used for the statistical analyses. Analysis of variance (ANOVA) was used for the inter- and intragroup analyses. In addition, Cohen's *d* test was used to indicate the treatment difference/effect size between two means (comparison between Po-Pr, Fo-Pr, and Fo-Po) within the groups [33, 34]. The outcome measures were VAS and PPT scores to assess neck pain and muscle tenderness, respectively. The level of significance (*α*) was set at *p* < 0.05.

## 3. Results

The results of the statistical analysis for all variables are as follows. There was homogenous distribution of all male participants with respect to their age (between 25 and 38 years) among group A (mean = 32.47 years), group B (mean = 32.13 years), and group C (mean = 32.33 years). Baseline measurements for both neck pain (VAS-Pr) and muscle tenderness, i.e., pressure pain threshold (PPT-Pr), showed insignificant differences (*p* > 0.05) among all groups as described in Table 1.

ANOVA calculated significant differences (*F*-values and *p* value) between and within groups for VAS-Po (*F* = 13.88, *p*=0.0001), VAS-Fo (*F* = 222.35, *p*=0.0001), PPT-Po (*F* = 17.17, *p*=0.0001), and PPT-Fo (*F* = 147.70, *p*=0.0001) with insignificant differences for VAS-Pr (*F* = 0.14, *p* > 0.05) and PPT-Pr (*F* = 0.13, *p* > 0.05) as described in Table 1.

Furthermore, post hoc (LSD) analysis compared the differences between the groups for all variables as given in Table 2. For VAS-Po, a significant difference was noted between group A and group B (mean difference = −1.40; *p*=0.0002); group A and group C (mean difference=−1.80; *p*=0.0001); and group B and group C (mean difference = −0.40; *p*=0.0210). For VAS-Fo, a significant difference was found between group A and group B (mean difference = -1.53; *p*=0.0001); group A and group C (mean difference = −5.27; *p*=0.0001); and group B and group C (mean difference = −3.73; *p*=0.0001). Similarly, for PPT-Po, a significant difference was detected between group A and group B (mean difference = 0.53; *p*=0.0007); group A and group C (mean difference = 0.87; *p*=0.0001); and group B and group C (mean difference = 0. 34; *p*=0.0250). In addition, for PPT-Fo, a significant difference was revealed between group A and group B (mean difference = 1.33; *p*=0.0001); group A and group C (mean difference = 2.25; *p*=0.0001); and group B and group C (mean difference = 0. 92; *p*=0.0001).

Cohen's *d* test was applied to assess the immediate and short-term treatment effect sizes within all the three groups for its variables VAS and PPT as follows. Effect size was understood as being large (*d* = 0.8), medium (*d* = 0.5), and small (*d* = 0.2).

### 3.1. Immediate Effects (Difference between Pre- and Postintervention within Groups)

On neck pain (VAS-Po—VAS-Pr), the treatment effect size was the largest in group A (mean difference = 3.00, *p* < 0.01, *d* = 1.77); larger in group B (mean difference = 1.80; *p* < 0.01; *d* = 1.30); and the smallest in group C (mean difference = 1.33, *p* < 0.05, *d* = 1.09). On muscle tenderness (PPT-Po – PPT-Pr), the treatment effect size was the largest in group A (mean difference = 0.96, *p* < 0.01, *d* = 1.49); larger in group B (mean difference = 0.49, *p* < 0.01, *d* = 1.03); and the smallest in group C (mean difference = 0.14, *p* > 0.05, *d* = 0.32) as described in Table 3.

### 3.2. Short-Term Effects (Difference between Preintervention and 2-Week Follow-Up)

On neck pain (VAS-Fo—VAS-Pr), the treatment effect size was the largest in group A (mean difference=5.27, *p* < 0.01, *d* = 4.04); larger in group B (mean difference = 3.93, *p* < 0.01, *d* = 3.02); and the smallest in group C (mean difference = 0.13, *p* > 0.05, *d* = 0.11). Regarding muscle tenderness (PPT-Fo—PPT-Pr), the treatment effect size was the largest in group A (mean difference = 2.14, *p* < 0.01, *d* = 3.89); larger in group B (mean difference = 0.87, *p* < 0.01, *d* = 1.76); and the smallest in group C (mean difference = 0.07, *p* > 0.05, *d* = 0.15) as described in Table 3.

## 4. Discussion

This study was intended to determine the immediate and short-term effects of combination therapies on reducing neck pain and muscle tenderness in patients with upper trapezius active MTrPs. All participants in experimental groups A and B and control group C received their specified intervention plan. The results of intergroup revealed that experimental group A yielded the greatest improvement immediately after intervention (*F*_VAS-Po_ = 13.879, *p* < 0.05) as well as after the 2-week follow-up (*F*_VAS-Fo_ = 222.348, *p* < 0.05) for all variables. In addition, the intragroup results showed that all of the intervention plans yielded significant improvement immediately after intervention as well as after the 2-week follow-up except control group C for all variables excluding the mean differences between VAS-Pr and VAS-Po, which showed significant improvement (mean difference = 1.33, *p* < 0.05, *d* = 1.09).

The results of this study can be understood with the reports of previous studies declared by Kashyap et al. [23], Iqbal et al. [21, 22], Hong et al. [39], Martín-Pintado-Zugasti et al. [9], Benito-de-Pedro et al. [12], Nasb et al. [41], Hanten et al. [19], Chaitow [13], Fryer and Hodgson [3], Fernández-de-las-Peñas et al. [36], Cagnie et al. [16, 20], Capo-Juan et al. [17], and other researchers. The results achieved by these authors are similar to the results achieved in this study for the combination of two manual techniques (MET plus ICT) in the management of neck pain and muscle tenderness due to upper trapezius active MTrPs.

The concept of relief of neck pain and decreased muscle tenderness (trigger point sensitivity) by MET can be understood through its neurophysiological effect such as inhibitory Golgi tendon reflex and descending pathway of pain modulation theories, anti-inflammatory and vascular effects [13, 18, 35]. MET (isometric contraction of agonist muscles) induces inhibitory Golgi tendon reflex which results in the reflex relaxation of the antagonist muscles. At the same time, the mechanoreceptors available in the joint and muscles get activated which further leads to the excitation of sympathetic system via somatic afferent and activation of the periaqueductal gray matter (PEG) which regulate the descending pain modulation [35, 36]. Rhythmic muscle contraction in MET also affects the rate of lymphatic and blood flow that bring the changes in interstitial pressure and increase transcapillary blood flow. Vascular blood flow desensitizes the peripheral nociceptive chemical mediators such as cytokines. [18].

The effects of MET can be also explained through the concept of lengthening of muscle fibers, which would help dictate the length of the affected soft tissues [4, 5, 7]. Lewit and Simons revealed that muscle lengthening utilizing postisometric relaxation seems effective in decreasing the sensitivity of MTrPs pain without the use of vapocoolant spray [37]. Furthermore, there is evolving proof supporting the activation of agonist-antagonist inhibitory pathways with the application of manual intervention [36]. Hence, different mechanisms would probably act at the same time to reduce pain intensity and muscle tenderness due to active MTrPs. Recently, Faqih et al. (2019) conducted a study using MET in patients with postsurgical elbow stiffness and found that the application of MET immediately after postsurgical elbow brought a significant improvement in pain intensity (VAS scores), ROM, and functions (DASH scores) [35]. Kashyap et al. (2018) revealed that the MPR and the MET are equally effective in improving the VAS, PPT, NDI, and range of rotation scores among the participants with nonspecific neck pain due to MTrPs [23]. In addition, our findings have been supported by Iqbal and colleagues (2013), who worked on combination therapies including only male patients and reported that the positional release technique in combination with MET showed immediate and short-term effectiveness in reducing the intensity of neck pain (VAS scores) as well as improving muscle tenderness (PPT scores) and functional status of the neck (NDI scores) in male patients with upper trapezius active MTrPs [22].

The ICT can be described by the concept of the “barrier release” proposed by Lewit (1991), in which the therapist slowly applies pressure to the MTrPs until a conclusive increase in resistance is perceived, i.e., the barrier, which is usually sensed as not being painful by the subject [38]. Hong et al. (1993) proved that prime results in decreasing pain from MTrPs were found with compression techniques used on the deep soft tissue when matching conventional soft-tissue manipulation [39]. Furthermore, Martín-Pintado-Zugasti et al. (2015) revealed that the ICT is effective in reducing post-dry needling soreness intensity and duration when dealing with patients with latent MTrPs [9]. Benito-de-Pedro et al. (2019) conducted a study to assess the immediate effectiveness of both deep dry needling and ICT on PPT and skin temperature in subjects with the latent MTrPs of the triceps surae and reported that the ICT could be more effective in reducing the local mechanosensitivity immediately after the treatment of a latent MTrP [12]. Likewise, Cagnie et al. (2013 and 2015) revealed a significant improvement in the scores of VAS (neck and shoulder pain), PPT, ROM, and muscle strength when applying the ICT among office workers having MTrPs with moderately severe chronic pain. Further, reduction in VAS scores with no change in NDI scores was noticed at 6-month follow-up [16, 20]. Previously, Gemmell et al. (2008) found that ischemic compression is superior to sham ultrasound in immediately reducing pain intensity in patients with nonspecific neck pain and upper trapezius active trigger points [15]. Fryer and Hodgson (2005) have also concluded that ICT was better than the sham myofascial technique at reducing muscle tenderness in latent MTrPs in the upper trapezius muscle [3].

Thus, the above-mentioned studies revealed that the application of ICT may induce analgesia and improve muscle tenderness (trigger point sensitivity) by the following mechanism. The pressure treatments may cause pain relief as a result of reactive hyperemia in the MTrPs region or act a spinal reflex mechanism for relieving muscle spasms [39]. Local pressure may align sarcomere length in the affected MTrPs and thus reduce pain, while deep pressure could offer effective stretching and mobilization of the taut bands [40]. Fryer and Hodgson (2005) already proved that local muscle tenderness due to MTrPs decreased only because of a change in tissue sensitivity rather than any unintentional release of pressure by the practitioner [3]. Hence, it can be concluded that ICT might be useful for decreasing neck pain (VAS) and improving muscle tenderness (PPT) in patients with upper trapezius active MTrPs.

In addition, combination therapy including ICT has proven to be more effective than the ICT alone, which supports the result of our study. Nasb et al. (2019) reported that the combination of ICT with dry cupping for 4 weeks has shown more effectiveness than either ICT or dry cupping alone in the improvement of PPT, NDI, and ROM scores significantly [41]. Hanten et al. (2000) examined the efficacy of a home program containing ischemic compression followed by sustained stretching over active MTrPs. The results of their study clearly revealed that the combination of these techniques was more effective in decreasing the muscle tenderness due to MTrPs [19]. Similarly, in a previous study (Iqbal et al. 2010) the short-term effect of ICT was also noted when applied in combination with strain-counterstrain in terms of pain relief, muscle tenderness, and functional status of the neck due to upper trapezius active MTrPs [21], thus supporting the findings of our study.

The improvement in the control group is attributed to the effects caused by stretching and hot pack use. Stretching of the affected muscle is believed to be an integral part of trigger point therapy. Jaeger and Reeves (1986), who stated the efficiency of spray and stretch at reducing pain intensity and increasing the pressure pain threshold, point out that vapocoolant spray could not bring anesthesia in the subcutaneous tissues or muscle because of the tissue depth. Therefore, they suggested that it is the stretch that reduced the pain sensitivity of the trigger points rather than the spray, thus reinforcing the idea that muscle lengthening is the process that offers pain relief [42]. Travell and Simons also argued that the stretch is the mechanism of relief in spray and stretch. They postulated that decreasing MTrPs pain utilizing spray and stretch is due to elongation of the muscle to its full normal length [7]. The patient's active or passive stretching exercises at home are more beneficial when performed during or soon after the application of moist heat [4]. Moist heat tends to relax the underlying muscles and diminish the tension in the trigger point, thus decreasing referred pain and local tenderness in response to pressure [5].

Because group A received both manual techniques such as MET and ICT, followed by conventional interventions such as active stretching and hot water fomentation, the higher benefit in pain relief and muscle tenderness (increased pain pressure threshold) may be credited to the above mechanism described and reinforced by different previous studies [12, 14, 15, 18–23, 35, 39–43]. However, MET alone and active stretching exercises were effective in group B but significantly less than in group A.

### 4.1. Limitations

This study included only male participants. We proposed to conduct a similar study among females through collaboration with female researchers and compare the results with current study. For this reason, the result of this study cannot be generalized for the female population of the same conditions. In addition, there was lack of advanced technology for measuring either the force of muscle contraction or amount of pressure required to stretch the muscle fibers/compress the trigger points to neutralize the MTrP pain. Moreover, only the immediate and short-term effect of combined manual therapies was assessed on unilateral upper trapezius MTrPs pain and muscle tenderness. Therefore, the above-mentioned shortcomings should be addressed by conducting a study on long-term effectiveness (12-week follow-up) of these combination techniques in bilateral upper trapezius MTrPs pain and muscle tenderness using advanced tools such as isokinetic machine and finger pressure algometer to execute an accurate and definitive amount of muscle contraction and application of pressure, respectively.

## 5. Conclusion

This study validated our hypothesis and concluded that MET plus ICT is more efficacious than MET alone in reducing neck pain and muscle tenderness in male patients with upper trapezius active MTrPs. Its immediate and short-term effects established this combination therapy as a prime treatment plan in the clinical setting to counteract the neck pain and muscle tenderness due to active MTrPs.

The clinical relevance of our findings to practice is that MET plus ICT is highly effective in dismissing MTrPs pain within a very brief period of time, is cost effective, is noninvasive, and achieves relief without causing much pain.

## Figures and Tables

**Table 1:** Intergroup comparison of PPT (kg/cm^2^) and VAS (cm) scores.

Variables	Group A mean ± SD	Group B mean ± SD	Group C mean ± SD	*F*-value	*p* value
VAS-Pr	6.20 ± 1.71	6.40 ± 1.47	6.33 ± 1.27	0.139	0.870
VAS-Po	3.20 ± 1.67	4.60 ± 1.27	5.00 ± 1.17	13.879	0.0001^*∗∗*^
VAS-Fo	0.93 ± 0.69	2.47 ± 1.11	6.20 ± 1.13	222.348	0.0001^*∗∗*^
PPT-Pr	1.71 ± 0.48	1.65 ± 0.47	1.67 ± 0.46	0.134	0.875
PPT-Po	2.67 ± 0.77	2.15 ± 0.48	1.81 ± 0.41	17.166	0.0001^*∗∗*^
PPT-Fo	3.85 ± 0.63	2.52 ± 0.51	1.60 ± 0.36	147.700	0.0001^*∗∗*^

^*∗*^Significant at *p* ≤ 0.05. ^*∗∗*^Highly significant at *p* ≤ 0.01.

**Table 2:** Between-group analysis (LSD a posteriori test) of the variables PPT (kg/cm^2^) and VAS (cm) scores.

Dependent variable	(*I*) group	(*J*) group	Mean difference (*I*-*J*)	Std. error	Sig.
VAS-Po	Group A	Group B	−1.40000-^*∗*^	0.3588	0.0002
		Group C	−1.80000-^*∗*^	0.3588	0.0001
	Group B	Group C	−0.40000-^*∗*^	0.3588	0.0210
VAS-Fo	Group A	Group B	−1.53333-^*∗*^	0.2569	0.0001
		Group C	−5.26667-^*∗*^	0.2569	0.0001
	Group B	Group C	−3.73333-^*∗*^	0.2569	0.0001
PPT-Po	Group A	Group B	0.52667^*∗*^	0.1491	0.0007
		Group C	0.86667^*∗*^	0.1491	0.0001
	Group B	Group C	0.34000^*∗*^	0.1491	0.0250
PPT-Fo	Group A	Group B	1.33333^*∗*^	0.1318	0.0001
		Group C	2.25333^*∗*^	0.1318	0.0001
	Group B	Group C	0.92000^*∗*^	0.1318	0.0001

**Table 3:** Treatment effect size (Cohen's *d*) within the group for PPT (kg/cm^2^) and VAS (cm) scores.

Dependent variable	Group a			Group B			Group C		
	Mean difference	*p* value	Effect size (*d*)^	Mean difference	*p* value	Effect size (*d*)^	Mean difference	*p* value	Effect size (*d*)^
VAS (Po-Pr)	3.00	<0.01^*∗∗*^	1.77	1.80	<0.01^*∗∗*^	1.30	1.33	<0.05^*∗*^	1.09
VAS (fo-pr)	5.27	<0.01^*∗∗*^	4.04	3.93	<0.01^*∗∗*^	3.02	0.13	>0.05	0.11
PPT (Po-Pr)	0.96	<0.01^*∗∗*^	1.49	0.49	<0.01^*∗∗*^	1.03	0.14	>0.05	0.32
PPT(Fo-pr)	2.14	<0.01^*∗∗*^	3.89	0.87	<0.01^*∗∗*^	1.76	0.07	>0.05	0.15

^*∗*^Significant at *p* ≤ 0.05. ^*∗∗*^Highly significant at *p* ≤ 0.01. ^Treatment effect size (*d*): large if *d* = 0.8, medium if *d* = 0.5, small if *d* = 0.2[33].

## Data Availability

The dataset supporting the conclusions of this article is available through the corresponding author on reasonable request.

## Abbreviations

MPS: Myofascial pain syndrome
MTrP: Myofascial trigger point
VAS: Visual analog scale
PPT: Pressure pain threshold
MET: Muscle energy technique
ICT: Ischemic compression technique

## References

[1] Occhionero V., Korpinen L., Gobba F. (2014). Upper limb musculoskeletal disorders in healthcare personnel. *Ergonomics*.

[2] Wang S. Y., Liu L. C., Lu M. C., Koo M. (2015). Comparisons of musculoskeletal disorders among ten different medical professions in Taiwan: a nationwide, population-based study. *PLoS One*.

[3] Fryer G., Hodgson L. (2005). The effect of manual pressure release on myofascial trigger points in the upper trapezius muscle. *Journal of Bodywork and Movement Therapies*.

[4] Simons D. G., Travell J. G., Simons L. S. (1999). *Travell & Simons’ Myofascial Pain and Dysfunction: Upper Half of Body*.

[5] Simons D. G. (2002). Understanding effective treatments of myofascial trigger points. *Journal of Bodywork and Movement Therapies*.

[6] Hong C.-Z., Hsueh T.-C. (1996). Difference in pain relief after trigger point injections in myofascial pain patients with and without fibromyalgia. *Archives of Physical Medicine and Rehabilitation*.

[7] Gerwin R. D., Shannon S., Hong C. Z., Hubbard D., Gevirtz R. (1997). Interrater reliability in myofascial trigger point examination. *Pain*.

[8] Fernández-de-las-Peñas C., Dommerholt J. (2017). International consensus on diagnostic criteria and clinical considerations of myofascial trigger points: a Delphi study. *Pain Medicine*.

[9] Martín-Pintado-Zugasti A., Pecos-Martin D., Rodríguez-Fernández Áodrí (20151). Ischemic compression after dry needling of a latent myofascial trigger point reduces postneedling soreness intensity and duration. *PM&R*.

[10] Gerwin R. D. (2014). Diagnosis of myofascial pain syndrome. *Physical Medicine and Rehabilitation Clinics of North America*.

[11] Calvo-Lobo C., Diez-Vega I., Martínez-Pascual B. (2017). Tensiomyography, sonoelastography, and mechanosensitivity differences between active, latent, and control low back myofascial trigger points: a cross-sectional study. *Medicine*.

[12] Benito-de-Pedro M., Becerro-de-Bengoa-Vallejo R., Losa-Iglesias M. E. (2019). Effectiveness between dry needling and ischemic compression in the triceps surae latent myofascial trigger points of triathletes on pressure pain threshold and thermography: a single blinded randomized clinical trial. *Journal of Clinical Medicine*.

[13] Chaitow L. (2015). *Positional Release Techniques*.

[14] Hou C.-R., Tsai L.-C., Cheng K.-F., Chung K.-C., Hong C.-Z. (2002). Immediate effects of various physical therapeutic modalities on cervical myofascial pain and trigger-point sensitivity. *Archives of Physical Medicine and Rehabilitation*.

[15] Gemmell H., Miller P., Nordstrom H. (2008). Immediate effect of ischaemic compression and trigger point pressure release on neck pain and upper trapezius trigger points: a randomised controlled trial. *Clinical Chiropractic*.

[16] Cagnie B., Castelein B., Pollie F., Steelant L., Verhoeyen H., Cools A. (2015). Evidence for the use of ischemic compression and dry needling in the management of trigger points of the upper trapezius in patients with neck pain. *American Journal of Physical Medicine & Rehabilitation*.

[17] Capo-Juan M. A. (2015). Cervical myofascial pain syndrome. Narrative review of physiotherapeutic treatment. *An Sist Sanit Navar*.

[18] Fryer G. (2011). Muscle energy technique: an evidence-informed approach. *International Journal of Osteopathic Medicine*.

[19] Hanten W. P., Olson S. L., Butts N. L., Nowicki A. L. (2000). Effectiveness of a home program of ischemic pressure followed by sustained stretch for treatment of myofascial trigger points. *Physical Therapy*.

[20] Cagnie B., Dewitte V., Coppieters I., Van Oosterwijck J., Cools A., Danneels L. (2013). Effect of ischemic compression on trigger points in the neck and shoulder muscles in office workers: a cohort study. *Journal of Manipulative and Physiological Therapeutics*.

[21] Iqbal A., Khan S. A., Miraj M. (2010). Efficacy of ischaemic compression technique in combination with strain-counterstrain technique in managing upper trapezius myofascial trigger point pain. *The Indian Journal of Physiotherapy & Occupational Therapy*.

[22] Iqbal A., Ahmed H., Shaphe A. (2013). Efficacy of muscle energy technique in combination with strain-counterstrain technique on deactivation of trigger point pain. *Indian Journal of Physiotherapy and Occupational Therapy—An International Journal*.

[23] Kashyap R., Iqbal A., Alghadir A. H. (2018). Controlled intervention to compare the efficacies of manual pressure release and the muscle energy technique for treating mechanical neck pain due to upper trapezius trigger points. *Journal of Pain Research*.

[24] Fernández-de-las-Peñas C., Alonso-Blanco C., Miangolarra J. C. (2007). Myofascial trigger points in subjects presenting with mechanical neck pain: a blinded, controlled study. *Manual Therapy*.

[25] Wolfe F., Clauw D. J., Fitzcharles M.-A. (2010). The American College of Rheumatology preliminary diagnostic criteria for fibromyalgia and measurement of symptom severity. *Arthritis Care & Research*.

[26] Fischer A. A. (1987). Pressure algometry over normal muscles. Standard values, validity and reproducibility of pressure threshold. *Pain*.

[27] Hogeweg J. A., Langereis M. J., Bernards A. T., Faber J. A., Helders P. J. (1992). Algometry. Measuring pain threshold, method and characteristics in healthy subjects. *Scandinavian Journal of Rehabilitation Medicine*.

[28] Vanderweeën L., Oostendorp R. A. B., Vaes P., Duquet W. (1996). Pressure algometry in manual therapy. *Manual Therapy*.

[29] Jensen M. P., Turner J. A., Romano J. M., Fisher L. D. (1999). Comparative reliability and validity of chronic pain intensity measures. *Pain*.

[30] Hawker G. A., Mian S., Kendzerska T., French M. (2011). Measures of adult pain: Visual Analog Scale for Pain (VAS Pain), numeric Rating Scale for Pain (NRS Pain), McGill Pain Questionnaire (MPQ), Short-Form McGill Pain Questionnaire (SF-MPQ), Chronic Pain Grade Scale (CPGS), Short Form-36 Bodily Pain Scale (SF). *Arthritis Care & Research*.

[31] Koo T. K., Guo J.-Y., Brown C. M. (2013). Test-retest reliability, repeatability, and sensitivity of an automated deformation-controlled indentation on pressure pain threshold measurement. *Journal of Manipulative and Physiological Therapeutics*.

[32] Alghadir A., Anwer S., Iqbal A., Iqbal Z. (2018). Test–retest reliability, validity, and minimum detectable change of visual analog, numerical rating, and verbal rating scales for measurement of osteoarthritic knee pain. *Journal of Pain Research*.

[33] Cohen J. (1988). *Statistical Power Analysis for the Behavioral Sciences*.

[34] Cohen J. (1992). Statistical power analysis. *Current Directions in Psychological Science*.

[35] Faqih A. I., Bedekar N., Shyam A., Sancheti P. (2019). Effects of muscle energy technique on pain, range of motion and function in patients with post-surgical elbow stiffness: a randomized controlled trial. *Hong Kong Physiotherapy Journal*.

[36] Fernández de las Peñas C., Sohrbeck Campo M., Fernández Carnero J., Miangolarra Page J. C. (2005). Manual therapies in the myofascial trigger point treatment: a systematic review. *Journal of Bodywork and Movement Therapies*.

[37] Lewit K., Simons D. G. (1984). Myofascial pain: relief by post-isometric relaxation. *Archives of Physical Medicine and Rehabilitation*.

[38] Lewit K. (1991). *Manipulative Therapy in Rehabilitation of the Locomotor System*.

[39] Hong C.-Z., Chen Y.-C., Pon C. H., Yu J. (1993). Immediate effects of various physical medicine modalities on pain threshold of an active myofascial trigger point. *Journal of Musculoskeletal Pain*.

[40] Blikstad A., Gemmell H. (2008). Immediate effect of activator trigger point therapy and myofascial band therapy on non-specific neck pain in patients with upper trapezius trigger points compared to sham ultrasound: a randomised controlled trial. *Clinical Chiropractic*.

[41] Nasb M., Qun X., Withanage C. R., Lingfeng X., Hong C. (2019). Dry cupping, ischemic compression, or their combination for the treatment of trigger points: a pilot randomized trial. *The Journal of Alternative and Complementary Medicine*.

[42] Jaeger B., Reeves J. L. (1986). Quantification of changes in myofascial trigger point sensitivity with the pressure algometer following passive stretch. *Pain*.

[43] Zamani S., Okhovatian F., Naimi S., Akbarzadeh A. (2017). The immediate effect of a combination of pressure release and cervical mobilization techniques on the active range of motion in the latent trigger points of upper trapezius muscle in young adult females. *Austin Physical Medicine*.

